# Uterine epithelioid leiomyosarcoma with c-kit expression and *YWHAE* gene rearrangement: a case report of a diagnostic pitfall of uterine sarcoma

**DOI:** 10.1186/s13000-017-0615-6

**Published:** 2017-03-14

**Authors:** Terufumi Kubo, Shintaro Sugita, Ryuichi Wada, Noriaki Kikuchi, Masahiro Iwasaki, Yumika Ito, Taro Sugawara, Hiromi Fujita, Makoto Emori, Ryoichi Tanaka, Hiroshi Hirano, Tsuyoshi Saito, Tadashi Hasegawa

**Affiliations:** 1 Department of Surgical Pathology, School of Medicine, Sapporo Medical University, South 1, West 16, Chuo-ku, Sapporo, Hokkaido 060-8543 Japan; 2 Department of Integrated Diagnostic Pathology, Nippon Medical School, Tokyo, Japan; 3 Department of Obstetrics and Gynecology, School of Medicine, Sapporo Medical University, Sapporo, Japan; 4 Department of Orthopaedic Surgery, School of Medicine, Sapporo Medical University, South 1, West 16, Chuo-ku, Sapporo, Hokkaido 060-8543 Japan

**Keywords:** *YWHAE* rearrangement, FISH, Uterine leiomyosarcoma, c-kit

## Abstract

**Background:**

Uterine sarcoma is a rare tumor that is often difficult to classify based on morphological and immunohistochemical analysis alone. Limited access to molecular biological analysis in routine practice would hinder making a definitive diagnosis.

**Case Presentation:**

In this report, we describe a case of a mesenchymal tumor arising from the uterine cervix in a 52-year-old woman. From microscopic morphology of the resected specimen, epithelioid leiomyosarcoma, high-grade endometrial stromal sarcoma, or uterine gastrointestinal stromal tumor (GIST) were considered as differential diagnoses. The immunophenotype of the tumor featured smooth muscle differentiation and hormone receptor expression. The cell membrane and cytoplasm were positive for c-kit, although no mutation was found in the *c-kit* or *PDGFRA* gene. Fluorescence *in situ* hybridization (FISH) analysis revealed a relatively low frequency of *YWHAE* rearrangement, whereas there were few *NUTM2A* and *NUTM2B* split signals.

**Conclusions:**

In this case, the tumor was not typical of any three of the differential diagnoses mentioned above. However, insufficient frequency of *YWHAE*, *NUTM2A,* and *NUTM2B* gene rearrangement and absence of mutation in both the *c-kit* and *PDGFRA* genes suggested that this tumor should be categorized as epithelioid leiomyosarcoma. This is an instructive case showing a potential diagnostic pitfall of uterine sarcoma. Comprehensive approaches including molecular biological techniques are required for definitive diagnosis.

## Background

Uterine sarcoma is a rare tumor for which diagnosis is often difficult. According to the 2014 WHO classification, uterine sarcoma consists of leiomyosarcoma, low- and high-grade endometrial stromal sarcoma (ESS), and undifferentiated sarcoma [1]. Uterine gastrointestinal stromal tumor (GIST), although not listed in the WHO classification, is reported in some studies [2]. As newly identified gene rearrangements or immunophenotypes in uterine sarcomas may establish novel disease entities, detailed pathological investigation in each case is becoming increasingly important for appropriate evaluation of the tumor.

We encountered an unusual case of mesenchymal tumor with epithelioid morphology. The tumor arose from the uterine cervix exhibiting relatively low-frequency mitosis, smooth muscle differentiation, and c-kit expression. Furthermore, *YWHAE* gene rearrangement was detected by fluorescence *in situ* hybridization (FISH) in the tumor. These findings were suggestive of epithelioid leiomyosarcoma, high-grade ESS, or uterine GIST as differential diagnosis.

## Case Presentation

**Fig. 1:**
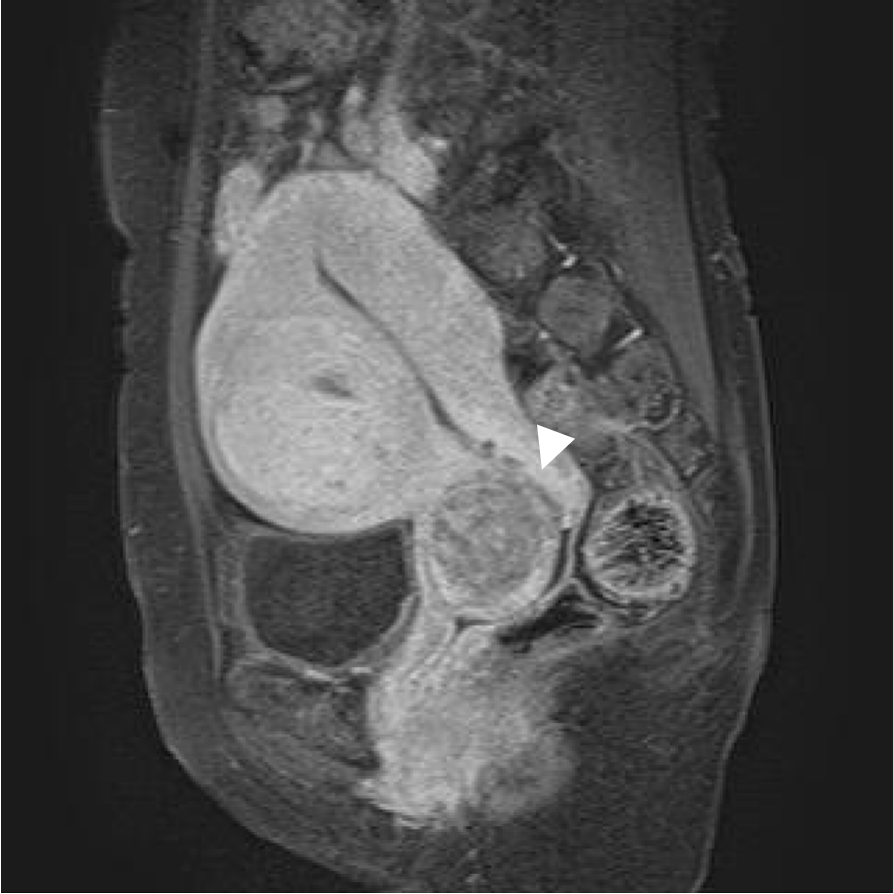
T1-weighted magnetic resonance imaging with fat suppression shows a low-signal lesion measuring approximately 4 cm at the uterine cervix (arrowhead)

A 52-year-old multiparous Japanese woman was referred to our hospital with a complaint of a feeling of abdominal fullness. She had uterine leiomyoma that had been observed for 9 years, and a history of chronic thyroiditis. Aside from the earlier observed leiomyoma, a previously unnoticed soft mass at the uterine cervix was palpable on pelvic examination. T1-weighted magnetic resonance imaging with fat suppression revealed a swollen uterine corpus with leiomyoma, and suggested a uterine cervical lesion with a low signal intensity (Fig. 1). Circulating levels of CA 125 and CA 19–9 were within the reference range at 16.3 and 11 U/mL, respectively. Hysterectomy was performed with a clinical diagnosis of multiple leiomyomas and an unknown cervical tumor. Postoperatively, the patient underwent adjuvant chemotherapy (gemcitabine plus docetaxel) and did well for the next 3 months, with neither local recurrence nor distant metastasis on chest and abdominal computed tomography imaging.

**Fig. 2:**
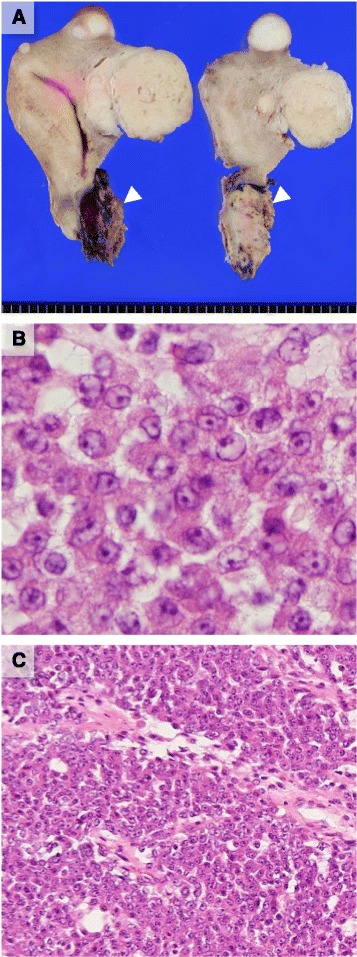
Gross and histopathological morphology of the uterine cervical lesion. **a** Cut surface of the resected uterus: Whitish masses in the uterine corpus are typical leiomyomas on gross and microscopic examination. At the uterine cervix, a gray-white lesion is observed (arrowhead). **b** High-power view of the lesion: Tumor cells have an epithelioid appearance but are less cohesive. Intercellular bridges are absent. Uniformly rounded nuclei have coarse chromatin and obvious nucleoli. Original magnification: ×400. **c** Low-power view of the lesion: Tumor cells are arranged in a nest-like structure compartmentalized by a vascular network. Original magnification: ×100

The enlarged uterus was 8 × 14 × 8.5 cm in size. Several elastic, hard, whitish masses were found in the uterine corpus, consistent with leiomyoma. In addition, an elastic, soft gray-white hemorrhagic mass measuring 8 × 6 × 5 cm was observed at the anterior wall of the uterine cervix (Fig. 2a).

Histologically, the cervical mass was below the squamous epithelium and consisted of a nest-like proliferation of less cohesive, epithelioid tumor cells that had rounded nuclei and eosinophilic cytoplasm. The tumor cells had no marked nuclear pleomorphism and prominent nucleoli, and a coarse chromatin pattern was found in the nucleus. Mitotic figures were moderately frequent (4 per high-power field) but atypical mitoses were not present (Fig. 2c). The tumor showed a confluent growth pattern and individual nests of tumor cells were compartmentalized by intricate vasculature (Fig. 2b). There was no apparent involvement of myometrial smooth muscle cells. Foci of extensive necrosis and hemorrhage were found in some areas. A low-grade ESS component, showing uniform cells with round to spindle-shaped nuclei, which were whorled around arteriole-type vessels, was not coexistent in the lesion.

**Fig. 3:**
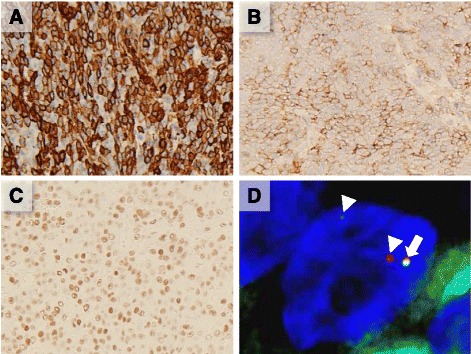
**a** to **c** Immunohistochemical analysis of the tumor. Tumor cells are positive for **a** α-smooth muscle actin, **b** c-kit, and **c** estrogen receptor. Original magnification: ×100. **d** Fluorescent *in situ* hybridization analysis: Arrow indicates non-split signal. Arrowheads indicate split signals. A split signal for the *YWHAE* gene is detected in 18% of tumor cells

Immunohistochemically, the tumor cells were diffusely positive for vimentin (clone V9, DAKO, Glostrup, Denmark), α-smooth muscle actin (αSMA; clone 1A4, DAKO; Fig. 3a), muscle-specific actin (clone HHF-35, DAKO), and heavy caldesmon (clone h-CD, DAKO), with moderate positivity for c-kit (DAKO; Fig. 3b), estrogen receptor (ER; clone SP1, Ventana, Tucson, AZ; Fig. 3c), and progesterone receptor (PgR; clone 1E2, Ventana). In contrast, the cells were negative for pan-cytokeratin (clone AE1/AE3, DAKO), desmin (clone D33, DAKO), CD10 (clone 56C6, Novocastra, Newcastle upon Tyne, United Kingdom), CD34 (clone QBEnd10, DAKO), p16 (clone E6H4, Roche, Basel, Switzerland), DOG1 (clone K9, Leica Biosystems, Wetzlar, Germany) and cyclin D1 (Biocare, Concord, CA). The Ki-67 (clone 30–9, Roche) labeling index of the neoplastic cells was 19.5%. These histological and immunohistochemical findings suggested a differential diagnosis of non-epithelial tumor of the uterus, particularly high-grade ESS, epithelioid leiomyosarcoma, or uterine GIST.

FISH was performed for further molecular biological observations. To detect *YWHAE* rearrangement derived from chromosomal translocation of t(10;17)(q22;p13)—we used a custom dual-color, split-signal *YWHAE* probe set for the *YWHAE* locus on chromosome 17p13 (Chromosome Science Lab, Inc., Sapporo, Japan). The probe set consisted of a 333-kilobase (kb) sequence labeled with SpectrumGreen (telomeric [RP11-143 L7 and RP11-22G12]) and a 303-kb sequence labeled with SpectrumOrange (centromeric [RP11-100 F18 and RP11-60C18]). Rearrangements in the *NUTM2A* and *NUTM2B* genes at 10q23 and 10q22, respectively, were characterized using a custom break-apart probe design. The probes were synthesized using oligo-based SureFISH technology (Agilent Technologies, Santa Clara, CA) and labeled with FITC (5’ probe) and Cy3 (3′ probe) fluorophores. The analysis was performed with formalin-fixed, paraffin-embedded specimens sectioned into 3-μm-thick slices, as described previously [3]. The split-signal rate *of YWHAE, NUTM2A* and *NUTM2B* was 18%, (Fig. 3d), 0 and 4%, respectively, when 50 nuclei were counted. In contrast, a *JAZF1* split, which is a major gene translocation in low-grade ESS, [4] was not detected.

To investigate *KIT* and *PDGFRA* gene mutations, direct sequence analysis was performed. Exons 9, 11, and 13 of the *KIT* gene and exon 18 of the *PDGFRA* gene were amplified by polymerase chain reaction using the primers KITexon9F: GGC TTT TGT TTT CTT CCC TTT A, KITexon9R: ATG GTA GAC AGA GCC TAA ACA, KITexon11F: GAT CTA TTT TTC CCT TTC TCC C, KITexon11R: AGC CCC TGT TTC ATA CTG AC, KITexon13F: GCT TGA CAT CAG TTT GCC AG, KITexon13R: GCA GCT TGG ACA CGG CTT T, PDGFRAexon18F: CAG ATG GCT TGA TCC TGA GT, and PDGFRAexon18R: GAG GAT GAG CCT GTC CAG T. The amplified products were purified and sequenced. The sequences of *KIT* exons 9, 11, and 13 were wild type. In *PDGFRA* exon 18, substitution of nucleotide C to T, known as a single-nucleotide variant (rs2228230) was detected in one allele. The variant does not cause amino acid substitution. Thus, it was considered that there was no oncogenic mutation in *KIT* and *PDGFRA* genes in this tumor.

## Discussion

This tumor consisted predominantly of round cells with a modest amount of eosinophilic to granular cytoplasm, irregular nuclear contour, and granular to vesicular chromatin with variably prominent nucleoli. Neoplastic cells were arranged in a nest-like structure compartmentalized by delicate vasculature. Although the current case conformed exactly to the morphological definition of high-grade ESS, the mitotic activity was inconspicuous. Immunohistochemically, c-kit and cyclin D1 are considered to be critical markers in diagnosing high-grade ESS [5, 6]. While the cell membrane and cytoplasm were positive for c-kit in the current case, there was no nuclear staining of cyclin D1. The diffuse positive signals for ER and PgR found in this case are also uncommon in high-grade ESS. The tumor cells were positive for some smooth muscle and myogenic markers including αSMA, muscle-specific actin, and heavy caldesmon, which have not been previously reported in high-grade ESS [7]. These immunophenotypes might indicate epithelioid leiomyosarcoma rather than high-grade ESS.

In addition to morphological and immunohistochemical findings, molecular biological investigations offer a sophisticated approach that provides critical information for accurately diagnosing some types of bone and soft tissue tumors. Lee et al. reported that high-grade ESS typically harbors a *YWHAE-NUTM2* fusion gene [8]. The current case harbored 18% of *YWHAE* gene rearrangement. Conversely, the frequencies of *NUTM2A* and *NUTM2B* split signals were far lower than that of *YWHAE*. Although a small number of split signals is considered to be sufficient evidence of gene rearrangement in the diagnosis for some types of tumors, a relevant threshold of *YWHAE* split signals for diagnosing high-grade ESS under the current concept is reported to be 20% [9]. Furthermore, *YWHAE* gene rearrangement may not be a specific finding for high-grade ESS. It was reported that translocation of t(10;17), including the *YWHAE* gene, was detected in a uterine sarcoma that was diagnosed as a poorly differentiated uterine tumor with t(10;17) translocation and neuroectodermal phenotype [10]. The c-kit positivity seen in the current case might possibly be suggestive of a uterine GIST. However, the results of direct sequencing revealed that there was no mutation in exons 9, 11, and 13 of the *KIT* gene or in exon 18 of the *PDGFRA* gene in the tumor. Based on all these findings, the tumor was designated as uterine epithelioid leiomyosarcoma with c-kit expression and *YWHAE* gene rearrangement.

## Conclusions

In conclusion, the tumor in this case seems quite instructive, showing a potential diagnostic pitfall of uterine sarcoma with epithelioid morphology. The tumor had some immunohistochemical features of leiomyosarcoma, high-grade ESS, and uterine GIST. In addition to the conventional practices for pathological diagnosis, molecular biological analysis led to a more definitive diagnosis. Interestingly, this uterine sarcoma exhibited unusual gene rearrangement for leiomyosarcoma. Accumulation of additional cases may enable an appropriate evaluation of prognosis, better treatment, and a novel paradigm for this disease.

## Abbreviations

ER: Estrogen receptor
ESS: Endometrial stromal sarcoma
FISH: Fluorescence *in situ* hybridization
GIST: Gastrointestinal stromal tumor
PgR: Progesterone receptor
